# Supplementary material for: Hematopoietic stem and progenitor cell membrane-coated vesicles for bone marrow-targeted leukaemia drug delivery
Source: Nat Commun. 2024 Jul 7;15:5689. doi: 10.1038/s41467-024-50021-9 (PMC11227508; doi:10.1038/s41467-024-50021-9)
Supplement: Supplementary file 1 — Supplementary Information [file 41467_2024_50021_MOESM1_ESM.pdf]

Supplementary Information

Hematopoietic stem and progenitor cell membrane-coated vesicles for bone marrow-targeted leukaemia drug delivery

Jinxin Li[1,2,3,#], Honghui Wu[4,5,6,#], Zebin Yu[1,2,3,#], Qiwei Wang[1,2,3,#], Xin Zeng[1,2,3,#], Wenchang Qian[1,2,3], Siqi Lu[1,2,3], Lingli Jiang[1,2,3], Jingyi Li[1,2,3], Meng Zhu[1,2,3], Yingli Han[1,2,3], Jianqing Gao[4,5,6,7,8,*], Pengxu Qian[1,2,3,*]

[1]Center for Stem Cell and Regenerative Medicine and Bone Marrow Transplantation Center of the First Affiliated Hospital, Zhejiang University School of Medicine, Hangzhou 310058, China.

[2]Liangzhu Laboratory, Zhejiang University, 1369 West Wenyi Road, Hangzhou 311121, China.

[3]Institute of Hematology, Zhejiang University & Zhejiang Engineering Laboratory for Stem Cell and Immunotherapy, Hangzhou 310058, China.

[4]Key Laboratory of Advanced Drug Delivery Systems of Zhejiang Province, College of Pharmaceutical Sciences, Zhejiang University, Hangzhou 310058, China

[5]Institute of Pharmaceutics, College of Pharmaceutical Sciences, Zhejiang University, Hangzhou 310058, PR China.

[6]Jinhua Institute of Zhejiang University, Jinhua, 321002, Zhejiang, PR China

[7]Department of Pharmacy, The Second Affiliated Hospital, Zhejiang University School of Medicine, Hangzhou, 310009, PR China

[8]Zhejiang University Cancer Center, Zhejiang University, Hangzhou 310058, PR China.

[#]These authors contributed equally to this work.

Corresponding authors:

*Pengxu Qian
Investigator
School of Medicine, Zhejiang University
Email: axu@zju.edu.cn

*Jianqing Gao
Professor
College of Pharmaceutical Sciences, Zhejiang University
Email: gaojianqing@zju.edu.cn

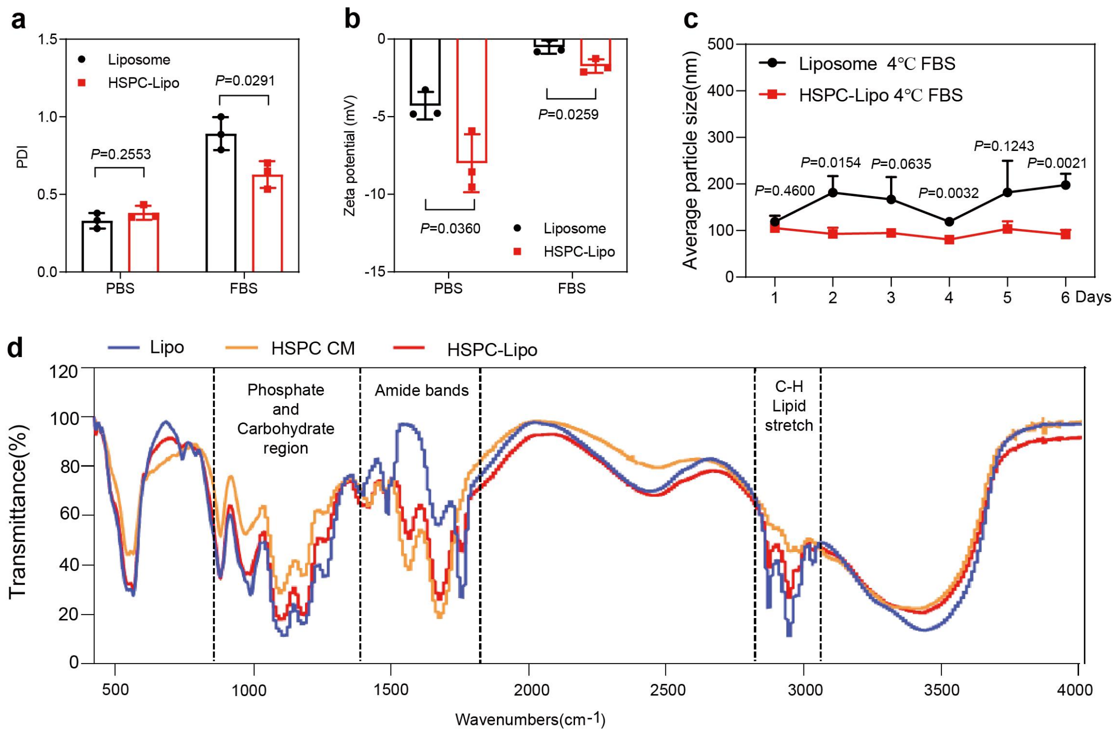

Supplementary Figure 1. Characterization of HSPC-Lipo. (**a**) Quantitative analysis of PDI and stability of HSPC-Lipo in FBS environment measured by DLS. Data were presented as mean ± s.d. n=3 experimental replicates. (**b**) Quantitative analysis of Zeta potential measured by DLS. Data were presented as mean ± s.d. n=3 experimental replicates. (**c**) Quantitative analysis of particle stability evaluation of HSPC-Lipo. Data were presented as mean ± s.d. n=3 experimental replicates. (**d**) Representative FTIR measurement image of liposome, HSPC cell membrane and HSPC-Lipo. The experiments were repeated 3 times independently with similar results. Source data are provided as a Source Data file.

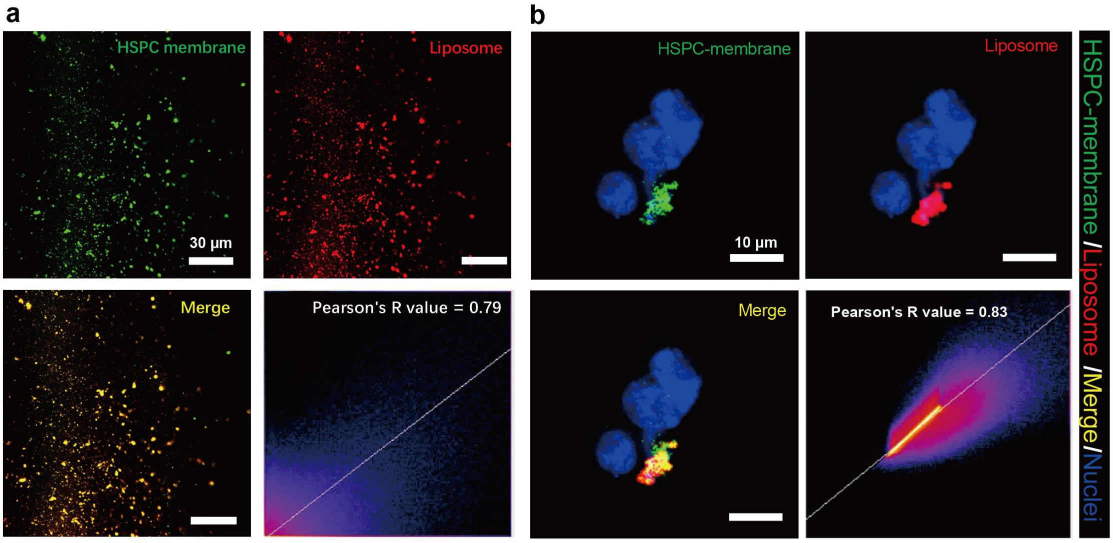

Supplementary Figure 2. The colocalization of HSPC membrane and liposomes. (**a**) Representative immunofluorescence images of HSPC membrane labeled by anti-CD44 antibody and secondary antibodies (Cy3-labeled Goat Anti-Rabbit IgG (H+L)) (represent in green). Liposomes were labeled by DiD dye at 25 μg mL^{-1} (represent in red). Scale bar, 30 μm. (**b**) Representative immunofluorescence images of HSPC-Lipo labeled by anti-CD44 antibody when combined with leukemia cells. Scale bar, 10 μm. The Pearson's R value was calculated by Fuji image J (version 153 with java 8). The experiments were repeated 3 times independently with similar results. Source data are provided as a Source Data file.

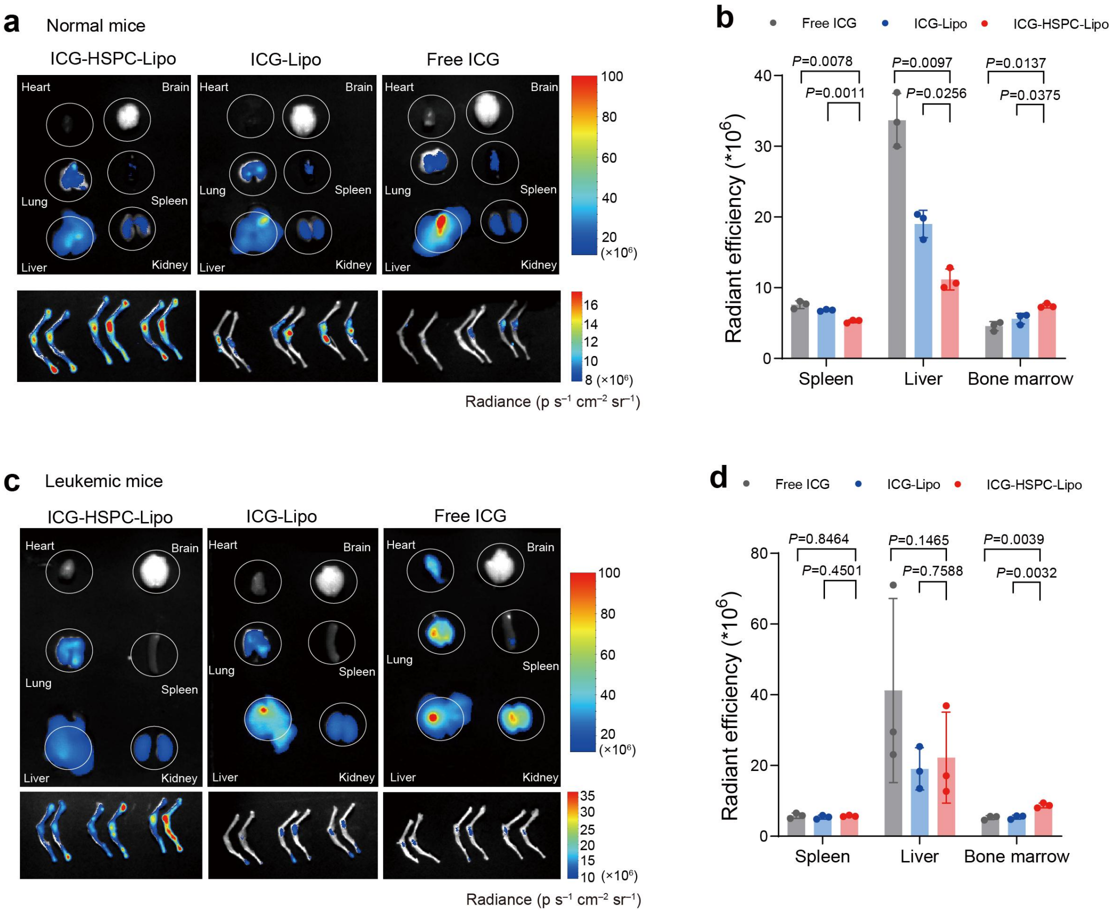

Supplementary Figure 3. The distribution of HSPC-Lipo in different organs after intravenous injection. (a) Representative fluorescence images of HSPC-Lipo in different organs of normal mice. Mice were euthanized for analysis at 24 hours after ICG labeled HSPC-Lipo was injected into the tail vein of mice. (**b**) Quantitative analysis of fluorescence in different organs of normal mice. Data were presented as mean ± s.d. (n=3 mice). **(c)** Representative fluorescence images of HSPC-Lipo in different organs of leukemic mice. Mice were euthanized for analysis at 24 hours after ICG labeled HSPC-Lipo was injected into the tail vein of mice. **(d)** Quantitative analysis of fluorescence in different organs of leukemic mice. The data were presented as mean ± s.d. (n=3 mice). Statistical significance of P values was calculated via a two-tailed, unpaired Student's t test and were indicated as * $P < 0.05$, ** $P < 0.01$ and *** $P < 0.001$. Source data are provided as a Source Data file.

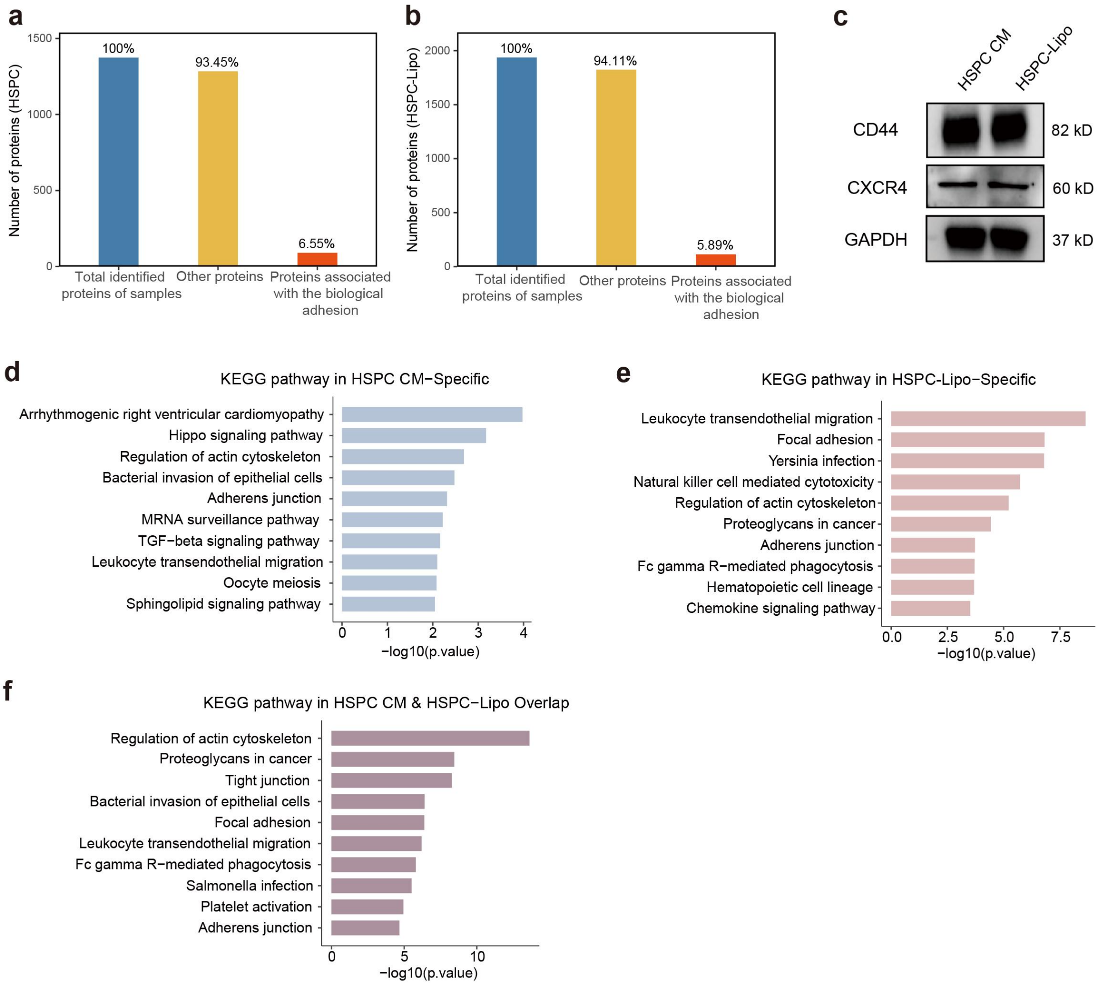

Supplementary Figure 4. Analysis and validation of adhesion related proteins of HSPC-Lipo nanoparticles. (a) The proportion of adhesion related proteins in HSPC cell membrane vesicle samples. **(b)** The proportion of adhesion related proteins in HSPC-Lipo samples. **(c)** Western blot analysis of CD44 and CXCR4 protein expression in HSPC membrane and HSPC-Lipo. The experiments were repeated 3 times independently with similar results. **(d-f)** KEGG pathway enrichment analysis of HSPC CM (**d**), HSPC-Lipo (**e**) and overlap of HSPC CM and HSPC-Lipo (**f**). The statistical analyses of P values were derived from a two-sided statistical test (**d-f**) and without adjustment for multiple comparisons. Source data are provided as a Source Data file.

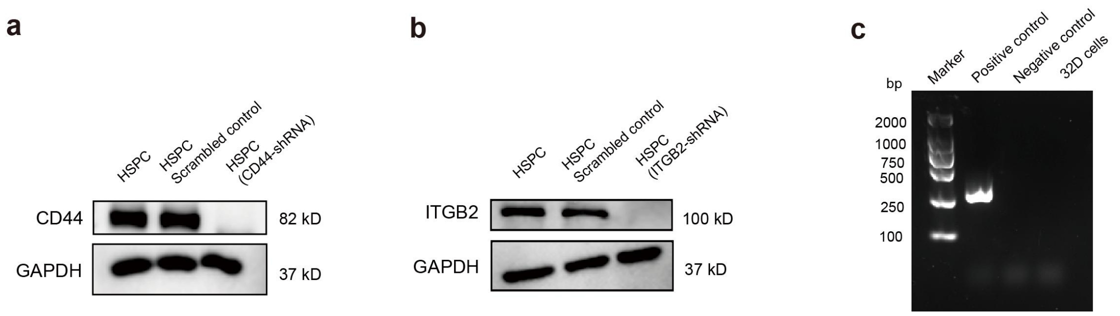

Supplementary Figure 5. Verification of the knockdown efficiency of CD44 and ITGB2 in mouse progenitor cell line (32D) using shRNA method. (a) Western blot analysis of CD44 protein knockdown efficiency. The experiments were repeated 3 times independently with similar results. (**b)** Western blot analysis of ITGB2 protein knockdown efficiency. The experiments were repeated 3 times independently with similar results. The ECL imaging and figures were collected by the Bio-Rad GelDoc Go with automatic exposure time. **(c)** The mycoplasma detection of progenitor cell line 32D cells. The experiments were repeated 3 times independently with similar results. Specific bands appearing at 280 bp were defined as mycoplasma contamination cells. Source data are provided as a Source Data file.

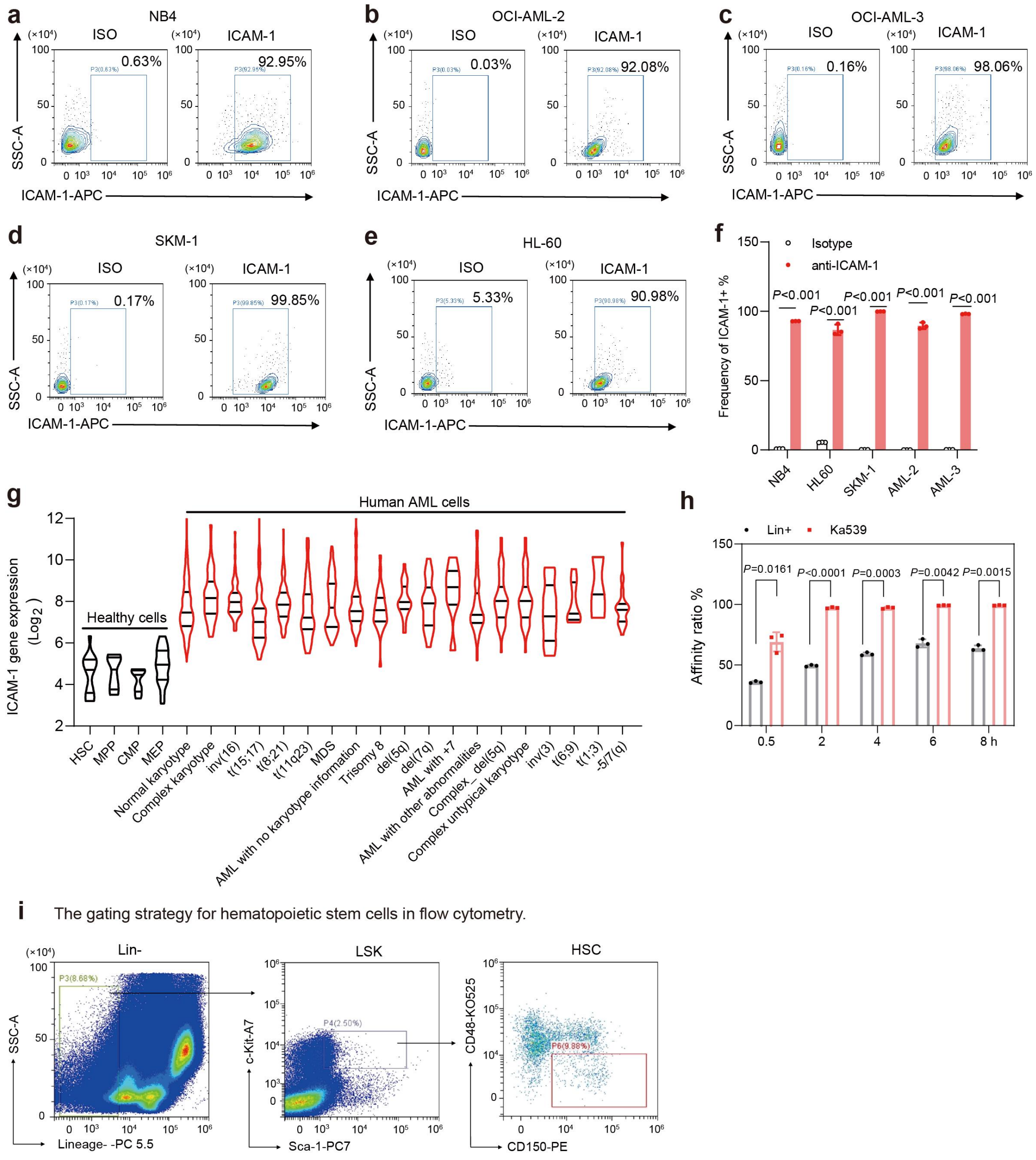

Supplementary Figure 6. Expression profile of ICAM-1 in leukemia cell lines and HSPC cells. (a-f) Representative flow cytometry plots of ICAM-1 expression in human leukemia cell lines. NB4 **(a)**, OCI-AML-2 **(b)**, OCI-AML-3 **(c)**, SKM-1 **(d)**, HL-60 **(e)**. **(f)** Quantitative analysis of ICAM-1expression in human leukemia cell lines. Data were presented as mean ± s.d. (n=3 experimental replicates). **(g)** Quantitative analysis of ICAM-1 expression (http://www.bloodspot.eu). **(h)** Quantitative analysis of affinity of HSPC-Lipo with leukemia cells and other lineage cells in bone marrow at different time point. Data were presented as mean ± s.d. (n=3 experimental replicates). **(i)** Representative flow cytometry plots of gating strategy of HSPC analysis. The data were presented as mean ± s.d. (n=3 experimental replicates). Statistical significance of P values was calculated via a two-tailed, unpaired Student's t test and were indicated as * P < 0.05, ** P < 0.01 and *** P < 0.001. Source data are provided as a Source Data file.

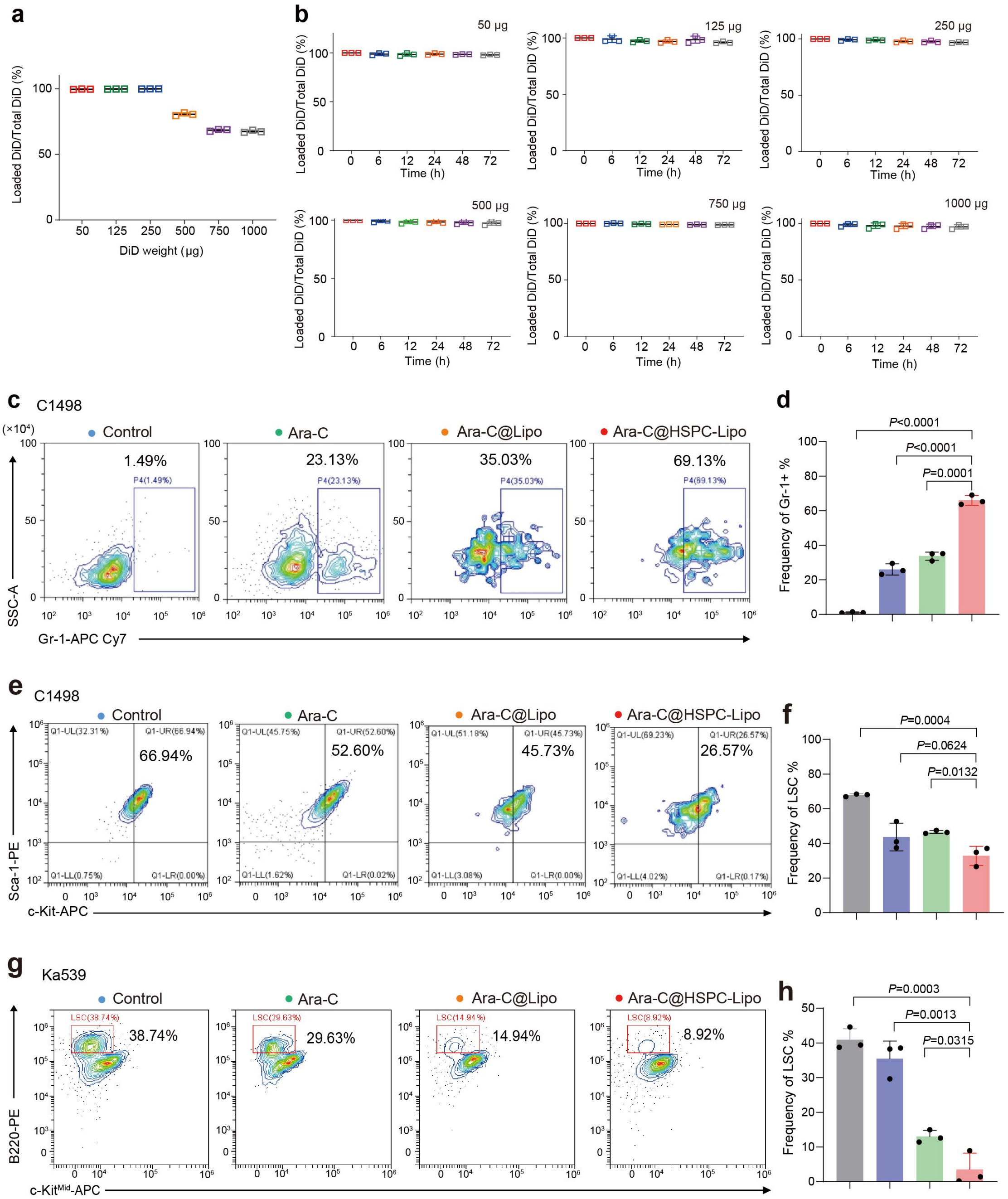

Supplementary Figure 7. DiD's encapsulation rate and in vitro cytotoxicity experiments at 72 hours after different treatments. (a-b) Quantitative analysis of DiD's encapsulation rate at different timepoint and DiD weights of 50, 125, 250, 500, 750 and 1000 μg. Data were presented as mean ± s.d. (n=3 experimental replicates). **(c)** Representative flow cytometry plots of cell differentiation of C1498 leukemia cells at 72h after different treatments. **(d)** Quantitative analysis of cell differentiation. Data were presented as mean ± s.d. (n=3 experimental replicates). **(e)** Representative flow cytometry plots of leukemia stem cells after different treatments at 72h in C1498 leukemia cells. **(f)** Quantitative analysis of leukemia stem cells in C1498 leukemia cells. Data were presented as mean ± s.d. (n=3 experimental replicates). **(g)** Representative flow cytometry plots of leukemia stem cells after different treatments

at 72h in Ka539 cells. **(h)** Quantitative analysis of leukemia stem cells in Ka539 cells. Data were presented as mean ± s.d. (n=3 experimental replicates). Statistical significance of P values was calculated via a two-tailed, unpaired Student's t test and were indicated as * $P < 0.05$, ** $P < 0.01$ and *** $P < 0.001$. Source data are provided as a Source Data file.

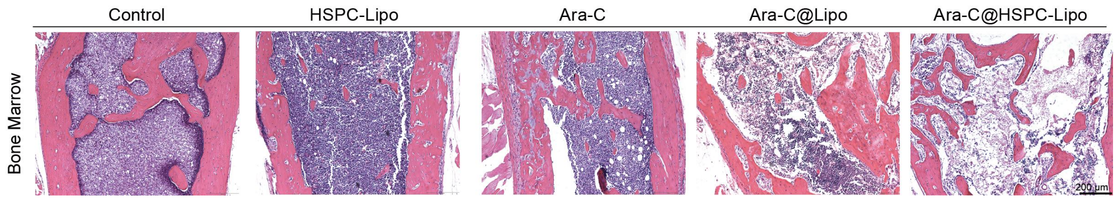

Supplementary Figure 8. H&E staining of BM in MLL-AF9 leukemia mouse model. Representative of H&E staining images of the infiltration of leukemia cells in BM after different treatment. Scale bars: 200 μm, respectively. The experiments were repeated 3 times independently with similar results.

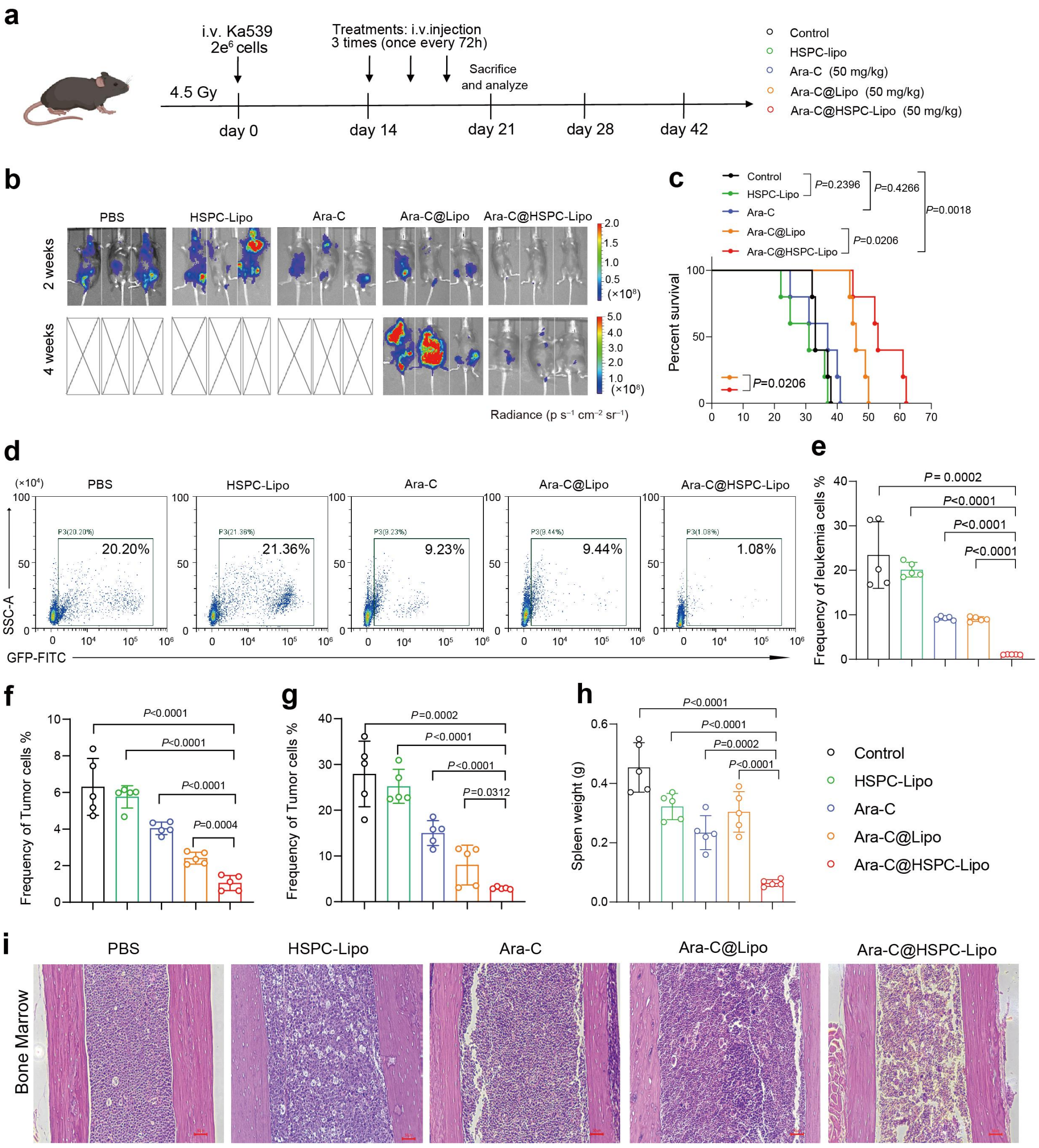

Supplementary Figure 9. The anti-leukemic effect of Ara-C@HSPC-Lipo in Ka539 leukemia mouse model. (a) Schematic illustration of animal experiment design. Cell membranes were derived from primary isolated HSPCs. Each mouse received approximately 20 μg of liposomes and 10 μg of cell membrane. **(b)** Representative fluorescence images of tumor burden after different treatments. **(c)** Survival curves of the leukemic mice received different treatments. The statistics and P-values were calculated using the Log-rank (Mantel-Cox) test. n=5 mice for each group. **(d)** Representative flow cytometry plots of leukemic cells (GFP positive cells) in bone marrow. Leukemic mice were euthanized at day 21. **(e)** Quantitative analysis of leukemic cells (GFP positive cells) in bone marrow. Data were presented as mean ± s.d. (n=5 mice). **(f)** Quantitative analysis of leukemic cells (GFP positive cells) in peripheral blood. Data were presented as mean ± s.d. (n=5 mice). **(g)** Quantitative analysis of leukemic cells (GFP positive cells) in spleen. Data were presented as mean

± s.d. (n=5 mice). **(h)** Spleen weight. Data represent the mean ± s.d. (n=5 mice). **(i)** Representative images of H&E staining of bone marrow sections. Scale bars, 500 μm. Statistical significance of P values was calculated via a two-tailed, unpaired Student's t test and were indicated as * $P < 0.05$, ** $P < 0.01$ and *** $P < 0.001$. Source data are provided as a Source Data file.

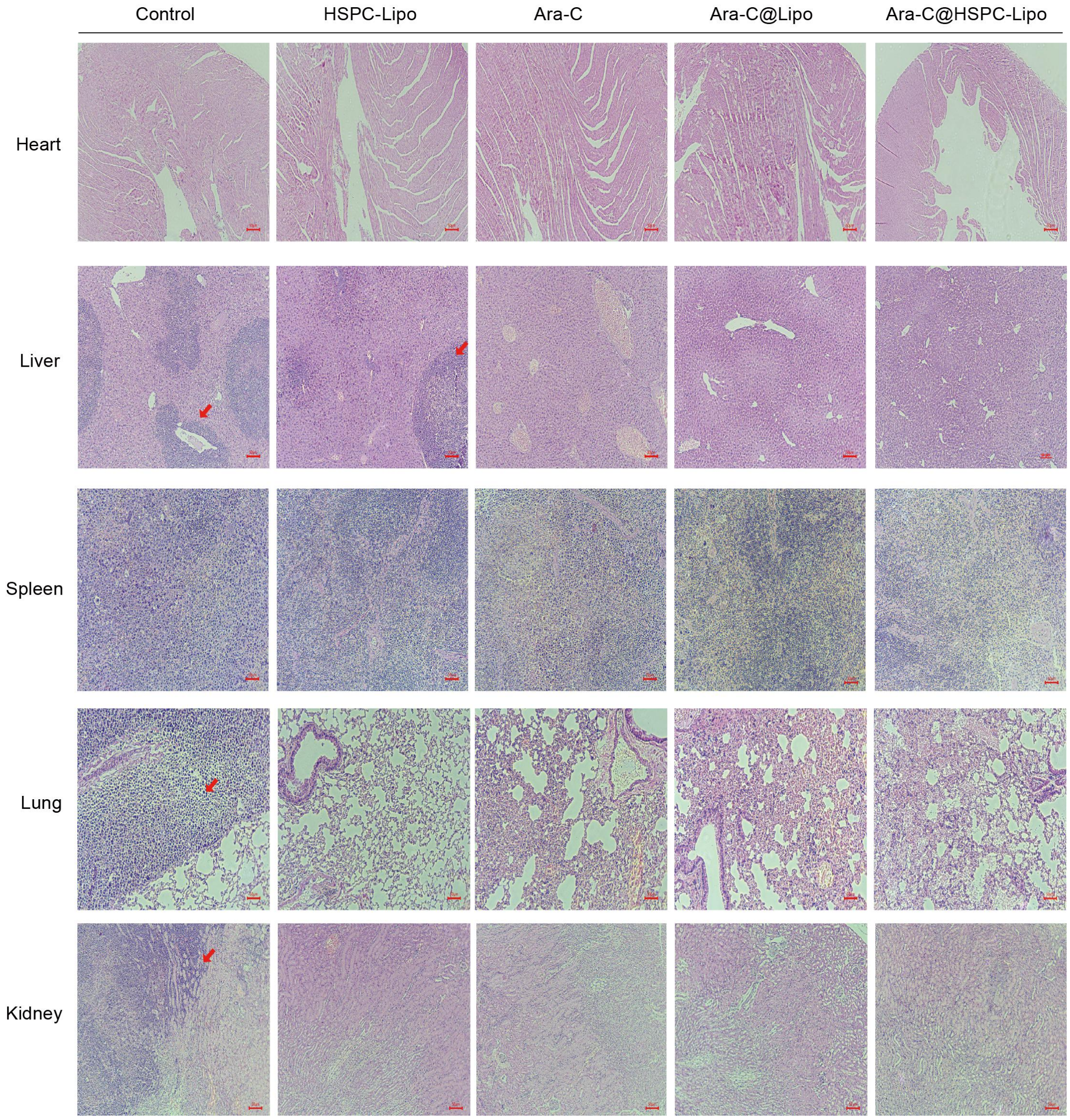

Supplementary Figure 10. H&E staining of main organs in Ka539 leukemia mouse model. Representative H&E staining images of the infiltration of tumor cells in different organs after Ara-C@HSPC-Lipo treatment. The red arrow indicates the tumor cell infiltration. Scale bars: 50 μm, respectively. The experiments were repeated 3 times independently with similar results.

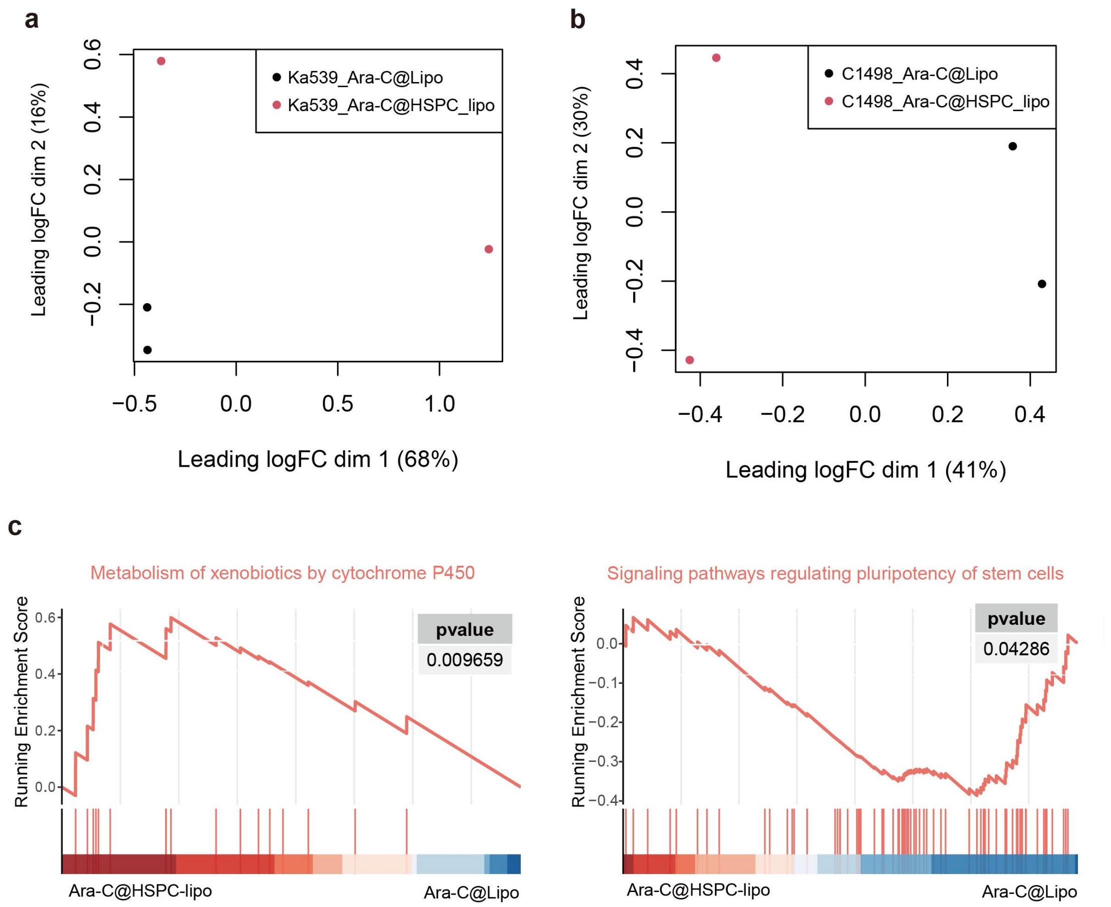

Supplementary Figure 11. Quality control of transcriptome data and GSEA analysis. (a) Principal Components Analysis of Ka539 cells after treatment with Ara-C@Lipo and Ara-C@HSPC-Lipo at 48 hours. (**b**) Principal Components Analysis of C1498 cells after treatment with Ara-C@Lipo and Ara-C@HSPC-Lipo at 48 hours. **(c)** GSEA enrichment analysis of Ka539 cells after treated with Ara-C@Lipo and Ara-C@HSPC-Lipo. The statistical analyses of P values were derived from a two-sided statistical test (**c**) and without adjustment for multiple comparisons.

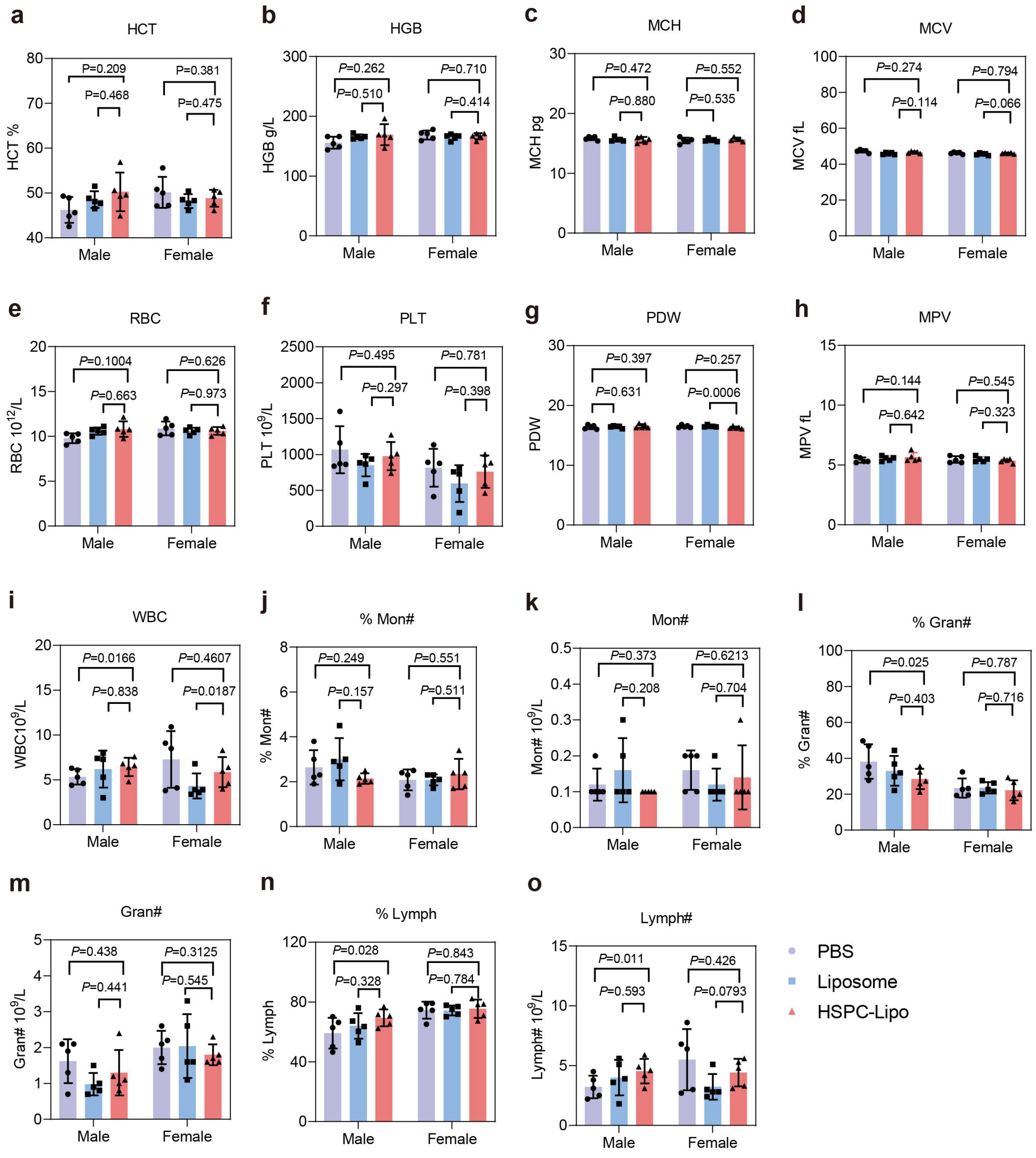

Supplementary Figure 12. Safety evaluation of hemogram in peripheral blood. (a-e) Quantitative analysis of Red blood cell related detection parameters. **(f-h)** Quantitative analysis of Platelet related detection parameters. **(i-o)** Quantitative analysis of White blood cell related detection parameters. The data represent the mean ± s.d. (**a-o**). n=5 mice (**a-o**). Statistical significance of P values was calculated via a two-tailed, unpaired Student's t test and were indicated as * $P < 0.05$, ** $P < 0.01$ and *** $P < 0.001$. Source data are provided as a Source Data file.

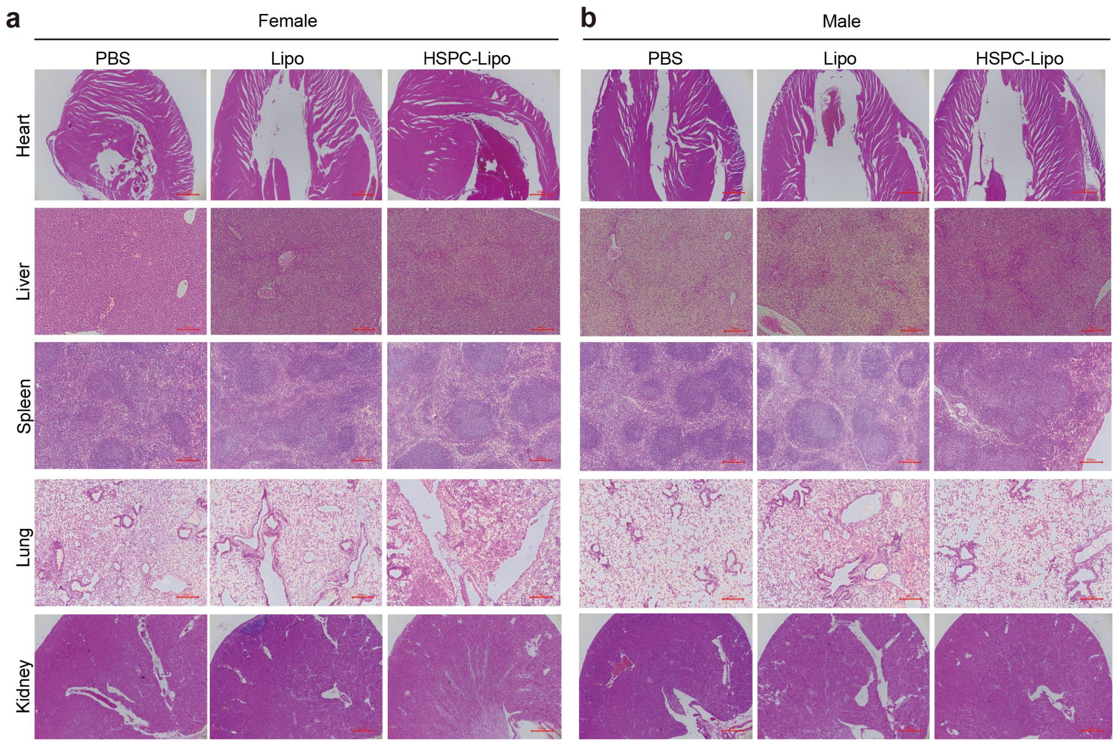

Supplementary Figure 13. Safety evaluation of H&E staining of organs. (a) Representative images of H&E staining of the main organs of female mice. Scale bars: 500 µm, respectively. (**b**) Representative images of H&E staining of the main organs of male mice. Scale bars: 500 µm, respectively. The experiments were repeated 3 times independently with similar results.

Supplementary Table 1. The proteins list in HSPCs cell membrane.

Protein names	Gene names
Desmoplakin (DP)	Dsp
Junction plakoglobin (Desmoplakin III) (Desmoplakin-3)	Jup
Serine/threonine-protein phosphatase PP1-beta catalytic subunit (PP-1B) (EC 3.1.3.16) (EC 3.1.3.53)	Ppp1cb
Serine/threonine-protein phosphatase 2A catalytic subunit alpha isoform (PP2A-alpha) (EC 3.1.3.16)	Ppp2ca
Vacuole membrane protein 1 (NF-E2-inducible protein 2) (Protein ni-2) (Transmembrane protein 49)	Vmp1 Tmem49
Catenin alpha-2 (Alpha N-catenin)	Ctnna2 Catna2
Cytoplasmic FMR1-interacting protein 2 (p53-inducible protein 121)	Cyfip2 Kiaa1168 Pir121
Ras-related C3 botulinum toxin substrate 1 (EC 3.6.5.2) (p21-Rac1)	Rac1
WD repeat-containing and planar cell polarity effector protein fritz homolog (mFrtz) (Homolog-13) (WD repeat-containing and planar cell polarity effector protein)	Wdpcp
Beta-1,3-galactosyl-O-glycosyl-glycoprotein beta-1,6-N-acetylglucosaminyltransferase (EC 2.4.1.102) (Core 2-branching enzyme) (Core2-GlcNAc-transferase) (C2GNT)	Gcnt1
Chordin	Chrd
Ras suppressor protein 1 (RSP-1) (Rsu-1)	Rsu1 Rsp1
Argininosuccinate synthase (EC 6.3.4.5) (Citrulline--aspartate ligase)	Ass1 Ass
E3 ubiquitin-protein transferase MAEA (EC 2.3.2.27) (Erythroblast macrophage protein) (Macrophage erythroblast attacher)	Maea Emp
Plakophilin-1	Pkp1
Switch-associated protein 70 (SWAP-70)	Swap70 Kiaa0640

Supplementary Table 2. The proteins list in HSPC-lipo.

Protein names	Gene names
Neuroplastin (Stromal cell-derived receptor 1) (SDR-1)	Nptn Sdfr1 Sdr1
DnaJ homolog subfamily A member 3, mitochondrial (DnaJ protein Tid-1) (mTid-1) (Tumorous imaginal discs protein Tid56 homolog)	Dnaja3 Tid1
Phosphatidylinositol 3-kinase regulatory subunit alpha (PI3-kinase regulatory subunit alpha) (PI3K regulatory subunit alpha) (PtdIns-3-kinase regulatory subunit alpha) (Phosphatidylinositol 3-kinase 85 kDa regulatory subunit alpha) (PI3-kinase subunit p85-alpha) (PtdIns-3-kinase regulatory subunit p85-alpha)	Pik3r1
Intercellular adhesion molecule 1 (ICAM-1) (MALA-2) (MyD10) (CD antigen CD54)	Icam1 Icam-1
AP-3 complex subunit beta-1 (Adaptor protein complex AP-3 subunit beta-1) (Adaptor-related protein complex 3 subunit beta-1) (Beta-3A-adaptin) (Clathrin assembly protein complex 3 beta-1 large chain)	Ap3b1
AP-3 complex subunit delta-1 (AP-3 complex subunit delta) (Adaptor-related protein complex 3 subunit delta-1) (Delta-adaptin) (mBLVR1)	Ap3d1 Ap3d
CD44 antigen (Extracellular matrix receptor III) (ECMR-III) (GP90 lymphocyte homing/adhesion receptor) (HUTCH-I) (Hermes antigen) (Hyaluronate receptor) (Lymphocyte antigen 24) (Ly-24) (Phagocytic glycoprotein 1) (PGP-1) (Phagocytic glycoprotein I) (PGP-I) (CD antigen CD44)	Cd44 Ly-24
Integrin alpha-5 (CD49 antigen-like family member E) (Fibronectin receptor subunit alpha) (Integrin alpha-F) (VLA-5) (CD antigen CD49e) [Cleaved into: Integrin alpha-5 heavy chain; Integrin alpha-5 light chain]	Itga5
Protein S100-A8 (Calgranulin-A) (Chemotactic cytokine CP-10) (Leukocyte L1 complex light chain) (Migration inhibitory factor-related protein 8) (MRP-8) (p8) (Pro-inflammatory S100 cytokine) (S100 calcium-binding protein A8)	S100a8 Caga Mrp8
Proteasomal ubiquitin receptor ADRM1 (110 kDa cell membrane glycoprotein) (Gp110) (Adhesion-regulating molecule 1) (ARM-1) (Rpn13 homolog)	Adrm1 Gp110
Signal transducer and activator of transcription 5B	Stat5b
Unconventional myosin-Ig	Myo1g
Zyxin	Zyx
Mammalian ependymin-related protein 1 (MERP-1)	Epdr1 Epdr2 Merp1 Merp2
Integrin alpha-IIb (GPalpha IIb) (GPIIb) (Platelet membrane glycoprotein IIb) (CD antigen CD41) [Cleaved into: Integrin alpha-IIb heavy chain; Integrin alpha-IIb light chain]	Itga2b
Fibrinogen gamma chain	Fgg
Ras-related C3 botulinum toxin substrate 2 (Protein EN-7) (p21-Rac2)	Rac2
Engulfment and cell motility protein 2 (Protein ced-12 homolog A)	Elmo2 Kiaa1834

Catenin delta-1 (Cadherin-associated Src substrate) (CAS) (p120 catenin) (p120(ctn)) (p120(cas))	Ctnnd1 Catns Kiaa0384
Tyrosine-protein phosphatase non-receptor type 11 (EC 3.1.3.48) (Protein-tyrosine phosphatase SYP) (SH-PTP2) (SHP-2) (Shp2)	Ptpn11
Rho-associated protein kinase 1 (EC 2.7.11.1) (Rho-associated, coiled-coil-containing protein kinase 1) (Rho-associated, coiled-coil-containing protein kinase I) (ROCK-I) (p160 ROCK-1) (p160ROCK)	Rock1
Unconventional myosin-If	Myo1f
Cysteine and glycine-rich protein 1 (Cysteine-rich protein 1) (CRP) (CRP1)	Csrp1 Crp1 Csrp
CD9 antigen (CD antigen CD9)	Cd9
Proto-oncogene vav (p95vav)	Vav1 Vav
F-actin-uncapping protein LRRC16A (CARMIL homolog) (Capping protein regulator and myosin 1 linker protein 1) (Capping protein, Arp2/3 and myosin-I linker homolog 1) (Capping protein, Arp2/3 and myosin-I linker protein 1) (CARML1) (Leucine-rich repeat-containing protein 16A)	Carmil1 Carmil Lrrc16 Lrrc16a
Synembryn-A (Protein Ric-8A)	Ric8a Ric8
Flotillin-1	Flot1
Alpha-actinin-1 (Alpha-actinin cytoskeletal isoform) (F-actin cross-linking protein) (Non-muscle alpha-actinin-1)	Actn1
Alpha-actinin-2 (Alpha-actinin skeletal muscle isoform 2) (F-actin cross-linking protein)	Actn2
ADP-ribosylation factor-like protein 2	Arl2
Very-long-chain 3-oxoacyl-CoA reductase (EC 1.1.1.330) (17-beta-hydroxysteroid dehydrogenase 12) (17-beta-HSD 12) (3-ketoacyl-CoA reductase) (KAR) (Estradiol 17-beta-dehydrogenase 12) (EC 1.1.1.62) (KIK-I)	Hsd17b12 Kik1
ADP-ribosylation factor 6	Arf6
Sialoadhesin (Sheep erythrocyte receptor) (SER) (Sialic acid-binding Ig-like lectin 1) (Siglec-1) (CD antigen CD169)	Siglec1 Sa Sn
Intercellular adhesion molecule 2 (ICAM-2) (Lymphocyte function-associated AG-1 counter-receptor) (CD antigen CD102)	Icam2 Icam-2
Integrin alpha-M (CD11 antigen-like family member B) (CR-3 alpha chain) (Cell surface glycoprotein MAC-1 subunit alpha) (Leukocyte adhesion receptor MO1) (CD antigen CD11b)	Itgam
Galectin-3-binding protein (Cyp-C-associated protein) (CyCAP) (Lectin galactoside-binding soluble 3-binding protein) (Protein MAMA)	Lgals3bp Cycap Mama
Myosin-binding protein C, fast-type (Fast MyBP-C) (C-protein, skeletal muscle fast isoform)	Mybpc2
GTP-binding protein 4 (Chronic renal failure gene protein) (GTP-binding protein NGB) (Nucleolar GTP-binding protein 1)	Gtpbp4 Crfg Nog1
Receptor-interacting serine/threonine-protein kinase 3 (EC 2.7.11.1) (RIP-like protein kinase 3) (Receptor-interacting protein 3) (RIP-3) (mRIP3)	Ripk3 Rip3

Supplementary Table 3. The proteins list in both cell membrane and HSPC-Lipo.

Protein names	Gene names
Adenosine deaminase (EC 3.5.4.4) (Adenosine aminohydrolase)	Ada
cAMP-dependent protein kinase type I-alpha regulatory subunit [Cleaved into: cAMP-dependent protein kinase type I-alpha regulatory subunit, N-terminally processed]	Prkar1a
Caspase-3 (CASP-3) (EC 3.4.22.56) (Apopain) (Cysteine protease CPP32) (CPP-32) (LICE) (Protein Yama) (SREBP cleavage activity 1) (SCA-1) [Cleaved into: Caspase-3 subunit p17; Caspase-3 subunit p12]	Casp3 Cpp32
Transforming protein RhoA (EC 3.6.5.2)	Rhoa Arha Arha2
Galectin-1 (Gal-1) (14 kDa lectin) (Beta-galactoside-binding lectin L-14-I) (Galaptin) (Lactose-binding lectin 1) (Lectin galactoside-binding soluble 1) (S-Lac lectin 1)	Lgals1 Gbp
Tubulin-specific chaperone D (Beta-tubulin cofactor D) (Tubulin-folding cofactor D)	Tbcd
Coronin-1C (Coronin-3)	Coro1c
Heat shock protein 105 kDa (42 degrees C-HSP) (Heat shock 110 kDa protein) (Heat shock-related 100 kDa protein E7I) (HSP-E7I)	Hsph1 Hsp105 Hsp110 Kiaa0201
Tyrosine-protein phosphatase non-receptor type 6 (EC 3.1.3.48) (70Z-SHP) (Hematopoietic cell protein-tyrosine phosphatase) (PTPTY-42) (Protein-tyrosine phosphatase 1C) (PTP-1C) (SH-PTP1) (SHP-1)	Ptpn6 Hcp Hcph Ptp1C
Leukosialin (B-cell differentiation antigen LP-3) (Leukocyte sialoglycoprotein) (Lymphocyte antigen 48) (Ly-48) (Sialophorin) (CD antigen CD43) [Cleaved into: CD43 cytoplasmic tail (CD43-ct) (CD43ct)]	Spn
Adenosine kinase (AK) (EC 2.7.1.20) (Adenosine 5'-phosphotransferase)	Adk
Transferrin receptor protein 1 (TR) (TfR) (TfR1) (Trfr) (CD antigen CD71)	Tfrc Trfr
Coronin-1A (Coronin-like protein A) (Clipin-A) (Coronin-like protein p57) (Tryptophan aspartate-containing coat protein) (TACO)	Coro1a Coro1
Band 3 anion transport protein (Anion exchange protein 1) (AE 1) (Anion exchanger 1) (MEB3) (Solute carrier family 4 member 1) (CD antigen CD233)	Slc4a1 Ae1
Spectrin alpha chain, erythrocytic 1 (Erythroid alpha-spectrin)	Spta1 Spna1 Spta
Purine nucleoside phosphorylase (PNP) (EC 2.4.2.1) (Inosine phosphorylase) (Inosine-guanosine phosphorylase)	Pnp Np Pnp1
Nck-associated protein 1-like (Hematopoietic protein 1)	Nckap1l Hem1
Fibronectin (FN) [Cleaved into: Anastellin]	Fn1
Radixin (ESP10)	Rdx
Tyrosine-protein kinase CSK (EC 2.7.10.2) (C-Src kinase) (Protein-tyrosine kinase MPK-2) (p50CSK)	Csk
Tyrosine-protein kinase SYK (EC 2.7.10.2) (Spleen tyrosine kinase)	Syk ptk72 Sykb

Integrin beta-2 (Cell surface adhesion glycoproteins LFA-1/CR3/p150,95 subunit beta) (Complement receptor C3 subunit beta) (CD antigen CD18)	Itgb2
Integrin beta-1 (Fibronectin receptor subunit beta) (VLA-4 subunit beta) (CD antigen CD29)	Itgb1
Cleft lip and palate transmembrane protein 1 homolog (Thymic epithelial cell surface antigen)	Clptm1 N14
Lactotransferrin (Lactoferrin) (EC 3.4.21.-)	Ltf
Galectin-9 (Gal-9)	Lgals9
Serine/threonine-protein phosphatase 2A 65 kDa regulatory subunit A alpha isoform (PP2A subunit A isoform PR65-alpha) (PP2A subunit A isoform R1-alpha)	Ppp2r1a
Gelsolin (Actin-depolymerizing factor) (ADF) (Brevin)	Gsn Gsb
40S ribosomal protein S3 (EC 4.2.99.18)	Rps3
Myosin-9 (Cellular myosin heavy chain, type A) (Myosin heavy chain 9) (Myosin heavy chain, non-muscle IIa) (Non-muscle myosin heavy chain A) (NMMHC-A) (Non-muscle myosin heavy chain IIa) (NMMHC II-a) (NMMHC-IIA)	Myh9
40S ribosomal protein SA (37 kDa laminin receptor precursor) (37LRP) (37 kDa oncofetal antigen) (37/67 kDa laminin receptor) (LRP/LR) (67 kDa laminin receptor) (67LR) (Laminin receptor 1) (LamR) (Laminin-binding protein precursor p40) (LBP/p40) (OFA/iLRP)	Rpsa Lamr1 P40-8
Glutathione S-transferase P 1 (Gst P1) (EC 2.5.1.18) (GST YF-YF) (GST class-pi) (GST-piB) (Preadipocyte growth factor)	Gstp1 Gstpib
Vinculin (Metavinculin)	Vcl
Cell division control protein 42 homolog (EC 3.6.5.2) (G25K GTP-binding protein)	Cdc42
CYFIP-related Rac1 interactor B (Protein FAM49B)	Cyrib Cyri Fam49b
Leukocyte surface antigen CD47 (Integrin-associated protein) (IAP) (CD antigen CD47)	Cd47
60 kDa heat shock protein, mitochondrial (EC 5.6.1.7) (60 kDa chaperonin) (Chaperonin 60) (CPN60) (HSP-65) (Heat shock protein 60) (HSP-60) (Hsp60) (Mitochondrial matrix protein P1)	Hspd1 Hsp60
Ras GTPase-activating-like protein IQGAP1	Iqgap1
Cofilin-1 (Cofilin, non-muscle isoform)	Cfl1
ATP synthase subunit beta, mitochondrial (EC 7.1.2.2) (ATP synthase F1 subunit beta)	Atp5f1b Atp5b
Protein RCC2	Rcc2 Kiaa1470
Growth factor receptor-bound protein 2 (Adapter protein GRB2) (SH2/SH3 adapter GRB2)	Grb2
Disintegrin and metalloproteinase domain-containing protein 10 (ADAM 10) (EC 3.4.24.81) (Kuzbanian protein homolog) (Mammalian disintegrin-metalloprotease) (CD antigen CD156c)	Adam10 Kuz Madm

Fermitin family homolog 3 (Kindlin-3) (Unc-112-related protein 2)	Fermt3 Kind3 Urp2
Ezrin (Cytovillin) (Villin-2) (p81)	Ezr Vil2
Alpha-actinin-4 (Non-muscle alpha-actinin 4)	Actn4
Chloride intracellular channel protein 1 (Nuclear chloride ion channel 27) (NCC27)	Clic1
Filamin-A (FLN-A) (Actin-binding protein 280) (ABP-280) (Alpha-filamin) (Endothelial actin-binding protein) (Filamin-1) (Non-muscle filamin)	Flna Fln Fln1
Guanine nucleotide-binding protein G(s) subunit alpha isoforms short (Adenylate cyclase-stimulating G alpha protein)	Gnas Gnas1 MNCb-5546
Protein disulfide-isomerase A6 (EC 5.3.4.1) (Thioredoxin domain-containing protein 7)	Pdia6 Txndc7
Talin-1	Tln1 Tln
Glia-derived nexin (GDN) (Peptidase inhibitor 7) (PI-7) (Protease nexin 1) (PN-1) (Protease nexin I) (Serine protease-inhibitor 4) (Serpin E2)	Serpine2 Pi7 Pn1 Spi4
Protein kinase C delta type (EC 2.7.11.13) (Tyrosine-protein kinase PRKCD) (EC 2.7.10.2) (nPKC-delta) [Cleaved into: Protein kinase C delta type regulatory subunit; Protein kinase C delta type catalytic subunit (Sphingosine-dependent protein kinase-1) (SDK1)]	Prkcd Pkcd
Amyloid beta A4 precursor protein-binding family B member 1-interacting protein (APBB1-interacting protein 1) (Proline-rich EVH1 ligand 1) (PREL-1) (Proline-rich protein 48)	Apbb1ip Prel1
Complement component 1 Q subcomponent-binding protein, mitochondrial (GC1q-R protein) (Glycoprotein gC1qBP) (C1qBP)	C1qbp Gc1qbp
Tropomyosin alpha-1 chain (Alpha-tropomyosin) (Tropomyosin-1)	Tpm1 Tpm-1 Tpma
Ras-related protein Rab-1A (EC 3.6.5.2) (YPT1-related protein)	Rab1A Rab1
Cyclin-dependent kinase 6 (EC 2.7.11.22) (CR2 protein kinase) (CRK2) (Cell division protein kinase 6) (Serine/threonine-protein kinase PLSTIRE)	Cdk6 Cdkn6 Crk2
Heat shock protein HSP 90-beta (Heat shock 84 kDa) (HSP 84) (HSP84) (Tumor-specific transplantation 84 kDa antigen) (TSTA)	Hsp90ab1 Hsp84 Hsp84-1 Hspcb
Calreticulin (CRP55) (Calregulin) (Endoplasmic reticulum resident protein 60) (ERp60) (HACBP)	Calr
Actin-related protein 2/3 complex subunit 2 (Arp2/3 complex 34 kDa subunit) (p34-ARC)	Arpc2
Dynamin-2 (EC 3.6.5.5) (Dynamin UDNM)	Dnm2 Dyn2
Polyunsaturated fatty acid lipoxygenase ALOX15 (12/15-lipoxygenase) (12/15-LO) (Arachidonate 12-lipoxygenase, leukocyte-type) (12-LOX) (L-12LO) (EC 1.13.11.31) (Arachidonate 15-lipoxygenase) (15-LOX) (EC 1.13.11.33) (Arachidonate omega-6 lipoxygenase) (Hepoxilin A3 synthase Alox15) (EC 1.13.11.-) (Linoleate 13S-lipoxygenase) (EC 1.13.11.12)	Alox15 Alox12l
Moesin (Membrane-organizing extension spike protein)	Msn
Heat shock protein HSP 90-alpha (EC 3.6.4.10) (Heat shock 86 kDa) (HSP 86)	Hsp90aa1

(HSP86) (Tumor-specific transplantation 86 kDa antigen) (TSTA)	Hsp86 Hsp86-1 Hspca
Neutrophil elastase (EC 3.4.21.37) (Elastase-2) (Leukocyte elastase)	Elane Ela2
Peroxiredoxin-2 (EC 1.11.1.24) (Thiol-specific antioxidant protein) (TSA) (Thioredoxin peroxidase 1) (Thioredoxin-dependent peroxide reductase 1) (Thioredoxin-dependent peroxiredoxin 2)	Prdx2 Tdpx1 Tpx
Gamma-parvin	Parvg
Myosin-10 (Cellular myosin heavy chain, type B) (Myosin heavy chain 10) (Myosin heavy chain, non-muscle IIb) (Non-muscle myosin heavy chain B) (NMMHC-B) (Non-muscle myosin heavy chain IIb) (NMMHC II-b) (NMMHC-IIB)	Myh10
Bystin	Bysl
Aminoacyl tRNA synthase complex-interacting multifunctional protein 1 (Multisynthase complex auxiliary component p43) [Cleaved into: Endothelial monocyte-activating polypeptide 2 (EMAP-2) (Endothelial monocyte-activating polypeptide II) (EMAP-II) (Small inducible cytokine subfamily E member 1)]	Aimp1 Emap2 Scye1
Phosphoglucomutase-like protein 5	Pgm5
Nucleoside diphosphate kinase B (NDK B) (NDP kinase B) (EC 2.7.4.6) (Histidine protein kinase NDKB) (EC 2.7.13.3) (P18) (nm23-M2)	Nme2
Beta-1,4-galactosyltransferase 1 (Beta-1,4-GalTase 1) (Beta4Gal-T1) (b4Gal-T1) (EC 2.4.1.-) (Beta-N-acetylglucosaminyl-glycolipid beta-1,4-galactosyltransferase) (Beta-N-acetylglucosaminylglycopeptide beta-1,4-galactosyltransferase) (EC 2.4.1.38) (Lactose synthase A protein) (EC 2.4.1.22) (N-acetyllactosamine synthase) (EC 2.4.1.90) (Nal synthase) (Neolactotriaosylceramide beta-1,4-galactosyltransferase) (EC 2.4.1.275) (UDP-Gal:beta-GlcNAc beta-1,4-galactosyltransferase 1) (UDP-galactose:beta-N-acetylglucosamine beta-1,4-galactosyltransferase 1) [Cleaved into: Processed beta-1,4-galactosyltransferase 1]	B4galt1 Ggtb Ggtb2

Supplementary Table 4. Encapsulation efficiency and loading capacity of HSPC-Lipo and HSPC membrane vesicles.

	Encapsulation efficiency (%)		Loading capacity (%)	
	HSPC-Lipo	HSPC membrane vesicle	HSPC-Lipo	HSPC membrane vesicle
Reverse-phase evaporation vesicle method	35.27±0.15	/	26.07±0.08	/
Film dispersion method	13.04±0.01	/	7.61±0.009	/
Incubation at room temperature method	10.24±0.005	0.96±0.004	7.20±0.003	0.65±0.004
pH gradient drug loading method	11.10±0.007	1.30±0.003	9.31±0.004	0.87±0.002

The Ara-C was loaded by different methods including reverse-phase evaporation vesicle method, film dispersion method, incubation at room temperature method and pH gradient drug loading method.
