# Supplementary material for: Hematopoietic stem and progenitor cell membrane-coated vesicles for bone marrow-targeted leukaemia drug delivery
Source: Nat Commun. 2024 Jul 7;15:5689. doi: 10.1038/s41467-024-50021-9 (PMC11227508; doi:10.1038/s41467-024-50021-9)
Supplement: Supplementary file 3 — Description of Additional Supplementary Files [file 41467_2024_50021_MOESM3_ESM.pdf]

<u>Description of Additional Supplementary Files</u>

Supplementary Data Legend:

Supplementary Data 1. The list of proteins detected by HSPC membrane and HSPC-Lipo.

Supplementary Data 2. The list of GO enrichment analysis results of the corresponding proteins.
