# Supplementary material for: Hematopoietic stem and progenitor cell membrane-coated vesicles for bone marrow-targeted leukaemia drug delivery
Source: Nat Commun. 2024 Jul 7;15:5689. doi: 10.1038/s41467-024-50021-9 (PMC11227508; doi:10.1038/s41467-024-50021-9)
Supplement: Supplementary file 6 — Reporting Summary [file 41467_2024_50021_MOESM6_ESM.pdf]

nature portfolio

Corresponding author(s): Pengxu Qian
Last updated by author(s): Jun 11, 2024

Reporting Summary

Nature Portfolio wishes to improve the reproducibility of the work that we publish. This form provides structure for consistency and transparency in reporting. For further information on Nature Portfolio policies, see our Editorial Policies and the Editorial Policy Checklist.

Statistics

For all statistical analyses, confirm that the following items are present in the figure legend, table legend, main text, or Methods section.

n/a	Confirmed	
☐	☒	The exact sample size (*n*) for each experimental group/condition, given as a discrete number and unit of measurement
☐	☒	A statement on whether measurements were taken from distinct samples or whether the same sample was measured repeatedly
☐	☒	The statistical test(s) used AND whether they are one- or two-sided<br>*Only common tests should be described solely by name; describe more complex techniques in the Methods section.*
☒	☐	A description of all covariates tested
☒	☐	A description of any assumptions or corrections, such as tests of normality and adjustment for multiple comparisons
☐	☒	A full description of the statistical parameters including central tendency (e.g. means) or other basic estimates (e.g. regression coefficient) AND variation (e.g. standard deviation) or associated estimates of uncertainty (e.g. confidence intervals)
☐	☒	For null hypothesis testing, the test statistic (e.g. *F*, *t*, *r*) with confidence intervals, effect sizes, degrees of freedom and *P* value noted<br>*Give P values as exact values whenever suitable.*
☒	☐	For Bayesian analysis, information on the choice of priors and Markov chain Monte Carlo settings
☒	☐	For hierarchical and complex designs, identification of the appropriate level for tests and full reporting of outcomes
☐	☒	Estimates of effect sizes (e.g. Cohen's *d*, Pearson's *r*), indicating how they were calculated

Our web collection on statistics for biologists contains articles on many of the points above.

Software and code

Policy information about availability of computer code

Data collection	1. TEM images were obtained with a Tecnai G2 F20 U-TWIN imaging system (FEI Company, USA).<br>2. The hydrodynamic size, PDI and zeta potential were determined by dynamic light scattering (Zetasizer Nano ZS, Malvern Instrument, UK).<br>3. FLow cytometry analysis was performed using Cytoflex LX (Beckman, Germany) .<br>4. The in vivo imaging was performed by small-animal imaging system (Maestro, Cambridge Research & Instrumentation, USA).<br>5. Immunofluorescence Images were taken with a confocal microscope OLYMPUS IX83-FV3000-OSR (Olympus, Japan).<br>6. Western Blot images were taken by Bio-Rad GelDoc Go system.<br>7.The RNA-seq data was performed by Illumina HiSeq 2500 (RNA-seq).<br>8. LC-MS/MS data was performed by a Q-Exactive HF X(Proteomics).<br>9. Blood biochemistries were measured with fully automatic biochemical analyser (Chemray 240, Shenzhen Rayto Life Science, China).<br>10. Hematologies were measured by automated hematology analyzer (Mindray, China).<br>11. The real-time PCR experients were performed on a Bio-rad PCR apparatus system.
Data analysis	1. The data statistical analysis were performed and graphed with Graphpad Prism 8.0.<br>2.The RNA-seg data were analyzed using Trimmomatic (v0.39), HISAT2 (v2.1.0), SAMtools (version 1.7), htseq-count (version 0.13.5), edgeR (v3.40.0), clusterProfiler package (version 4.7.1).<br>3. The proteomics data were analyzed using clusterProfiler package (version 4.7.1).<br>4. The fluorescence images were processed using ImageJ v2.0.0.<br>5. The flow cytometry data were analyzed by CytExpert 2.3 and Flowjo v10.

For manuscripts utilizing custom algorithms or software that are central to the research but not yet described in published literature, software must be made available to editors and reviewers. We strongly encourage code deposition in a community repository (e.g. GitHub). See the Nature Portfolio guidelines for submitting code & software for further information.

Data

Policy information about availability of data

All manuscripts must include a data availability statement. This statement should provide the following information, where applicable:

- Accession codes, unique identifiers, or web links for publicly available datasets
- A description of any restrictions on data availability
- For clinical datasets or third party data, please ensure that the statement adheres to our policy

The authors declare that all relevant raw data presented in main figures and supplementary figures has been provided in the Source Data file. Uncropped and unprocessed scans of blots have been provided as in the Source Data file. The raw data of RNA-seq have been deposited in the gene expression omnibus (GEO) repository under the accession number GEO: GSE232029 (https://www.ncbi.nlm.nih.gov/geo/query/acc.cgi?acc=GSE232029). The mass spectrometry proteomics data have been deposited to the ProteomeXchange Consortium via the iProX partner repository with the dataset identifier PXD052979 (https://proteomecentral.proteomexchange.org/cgi/GetDataset?ID=PXD052979). The protein mass spectrometry data generated in this study are provided in the Supplementary Data 1-2. Source data are provided with this paper.

Research involving human participants, their data, or biological material

Policy information about studies with human participants or human data. See also policy information about sex, gender (identity/presentation), and sexual orientation and race, ethnicity and racism.

Reporting on sex and gender	If this information has not been collected.
Reporting on race, ethnicity, or other socially relevant groupings	See above
Population characteristics	See above
Recruitment	This information has not been collected.
Ethics oversight	This information has not been collected.

Note that full information on the approval of the study protocol must also be provided in the manuscript.

Field-specific reporting

Please select the one below that is the best fit for your research. If you are not sure, read the appropriate sections before making your selection.

☒ Life sciences ☐ Behavioural & social sciences ☐ Ecological, evolutionary & environmental sciences

For a reference copy of the document with all sections, see nature.com/documents/nr-reporting-summary-flat.pdf

Life sciences study design

All studies must disclose on these points even when the disclosure is negative.

Sample size	Sample size was determined based on control samples and the experimental samples. Specifically, in the animal experiment, there were 6-12 mice in each group, acording to different specific experimental designs.
Data exclusions	No data were excluded from the analyses.
Replication	All in vitro experiments were replicated independently for at least 3 biologically independent experiments. In vivo sample size (n) in each group is detailed in the figure legends or methods section. All attempts at replication were successful.
Randomization	Throughout all studies, samples and animals were randomized into groups.
Blinding	Blinding was used in animal allocation,data collection and analysis.

Reporting for specific materials, systems and methods

We require information from authors about some types of materials, experimental systems and methods used in many studies. Here, indicate whether each material, system or method listed is relevant to your study. If you are not sure if a list item applies to your research, read the appropriate section before selecting a response.

Materials & experimental systems

n/a	Involved in the study
☐	☒ Antibodies
☐	☒ Eukaryotic cell lines
☒	☐ Palaeontology and archaeology
☐	☒ Animals and other organisms
☒	☐ Clinical data
☒	☐ Dual use research of concern
☒	☐ Plants

Methods

n/a	Involved in the study
☒	☐ ChIP-seq
☐	☒ Flow cytometry
☒	☐ MRI-based neuroimaging

Antibodies

Antibodies used

The antibodies used in flow cytometry (FCM) were as follow:
Ly-6G/Ly-6C Monoclonal Antibody (RB6-8C5, APC-Cyanine7, BioLegend, #108424)
Anti-mouse CD45 (BV510, BioLegend, #103138)
CD11b Antibody (M1/70, PE-Cyanine7, eBioscience, #25-0112-82)
CD3e Monoclonal Antibody (145-2C11, APC, eBioscience, #17-0031-83)
CD4 Monoclonal Antibody (RM4-5, PE-Cyanine5, eBioscience, #15-0042-83)
CD8a Monoclonal Antibody (53-6.7, PE-Cyanine5, eBioscience, #15-0081-83)
CD3e Monoclonal Antibody (145-2C11, PE-Cyanine5, eBioscience, #15-0031-83)
CD4 Monoclonal Antibody (RM4-5, PE-Cyanine5, eBioscience, #15-0042-83)
CD8a Monoclonal Antibody (53-6.7, PE-Cyanine5, eBioscience, #15-0081-83)
CD11b Monoclonal Antibody (M1/70, PE-Cyanine5, eBioscience, #15-0112-83)
Ly-6G/Ly-6C Monoclonal Antibody (RB6-8C5, PE-Cyanine5, eBioscience, #15-5931-83)
CD45R (B220) Monoclonal Antibody (RA3-6B2, PE-Cyanine5, eBioscience, #15-0452-83)
IgM Monoclonal Antibody (II/41, PE-Cyanine5, eBioscience, #15-5790-82)
TER-119 Monoclonal Antibody (TER-119, PE-Cyanine5, eBioscience, #15-5921-83)
Ly-6A/E (Sca-1) Monoclonal Antibody (D7, PE-Cyanine7, BioLegend, #108114)
CD117 (c-Kit) Monoclonal Antibody (2B8, APC, eBioscience, #17-1171-83)
anti-mouse CD150 (SLAM) Antibody (TC15-12F12.2, PE, BioLegend, #115904)
CD48 Monoclonal Antibody (HM48-1, eFluor 450, eBioscience, #48-0481-82)
Rat Anti-mouse CD34 (RAM34, Alexa Fluor R 647, BD Pharm, #560230)
Anti-mouse CD117 (c-kit, APC/cy7, BioLegend, #105826)
Ly-6A/E (Sca-1) Monoclonal Antibody (D7, PE, eBioscience, #12-5981-82)
CD127 Monoclonal Antibody (A7R34, eFluor 450, eBioscience, #48-1271-82)
CD16/CD32 Monoclonal Antibody (93, PE-Cyanine7, eBioscience, #25-0161-82)
Anti-human/mouse CD45R (B220) Antibody (RA3-6B2, PE, eBioscience, #12-0452-83)
Hamster IgG1, λ1 Isotype Control (Clone: G235-2356, BV510, BD Horizon, #562954)
Rat IgG2b kappa Isotype Control (APC-eFluor 780, eBioscience, #47-4031-82)
Rat IgG2a kappa Isotype Control (PE-Cyanine7, eBioscience, #25-4321-82)
Mouse IgG1 kappa Isotype Control (P3.6.2.8.1, APC, eBioscience, #17-4714-81)
Rat IgG2a kappa Isotype Control (PE, eBioscience, #12-4321-83)
Rat IgG2a kappa Isotype Control (PE-Cyanine5, eBioscience, #15-4321-82)
Armenian Hamster IgG Isotype Ctrl Antibody (PE, BioLegend, #400908)
Rat IgG1, k, Isotype Control (x40, BV421, BD Horizon, #562438)
Rat IgG2b kappa Isotype Control (APC-eFluor 780, eBioscience, #47-4031-82)
Hamster IgG1, λ1 Isotype Control (Clone: G235-2356, BV510, BD Horizon, #562954)

The antibodies used in western blot were as follow:
Anti-CD44 (Boster, Cat. A00052, overnight, dilution 1:1000)
Anti-CXCR4 (Beyotime, Cat. AF6621, overnight, dilution 1:1000)
Anti-ITGB2 (Beyotime, Cat. AF6399, overnight, dilution 1:1000)
Anti-GAPDH (Sangon Biotech, Cat. D190090-0100, overnight, dilution 1:5000)
Anti-HA (Cloud-Clone, US, Cat. PAA182Ge01, dilution 1:500)
Anti-ICAM-1(Beyotime, Cat. AF1774, dilution 1:1000)
Goat Anti-Mouse IgG(H+L)(peroxidase/HRP conjugated), Elabscience, Cat. E-AB-1001, dilution 1:5000)
Goat Anti-Rabbit IgG (H+L)(peroxidase/HRP conjugated), Elabscience, Cat. E-AB-1003, dilution 1:5000)

Validation

Validation of antibodies used in western blotting, immunofluorescence, and flow cytometry was performed by the suppliers (BD Bioscience, Biolegend, Boster, Beyotime, Sangon Biotech, Cloud-Clone and Elabscience). Relevant data are available on the manufacturer's official website. No further validation was performed.

Eukaryotic cell lines

Policy information about cell lines and Sex and Gender in Research

Cell line source(s)

The mouse cell lines C1498 (ATCC TIB-49) and 32D (ATCC CRL-3594) were obtained from the American Type Culture Collection (ATCC). The mouse cell line Ka539 was a gift from Y. Liu (Sichuan University). The human AML cell lines HL-60 (ATCC CCL-240) were obtained from the American Type Culture Collection (ATCC). The human AML cell lines OCI-AML2

	(DSMZ ACC 99), OCI-AML3 (DSMZ ACC 582), SKM-1 (DSMZ ACC 547) and NB-4 (DSMZ ACC 207) were purchased from the Leibniz-Institute DSMZ (http://www.dsmz.de).
Authentication	No further authentication was performed after receiving the cells as a gift or from ATCC
Mycoplasma contamination	Testing was performed by PCR. All cell lines tested negative for mycoplasma. All cell lines were routinely checked for Mycoplasma contamination using Hoechst staining and careful maintained.
Commonly misidentified lines (See ICLAC register)	No commonly misidentified cell lines were used.

Animals and other research organisms

Policy information about studies involving animals; ARRIVE guidelines recommended for reporting animal research, and Sex and Gender in Research

Laboratory animals	C57BL/6 mice, BALB/c mice and SD rats were purchased from The Jackson Laboratory, Gempharmatech Co., Ltd. and Animal Center, School of Medicine, Zhejiang University. Female mice aged 6 to 8 weeks were used for the experiment. All mice were kept in an SPF environment. All mice were kept under specific pathogen-free conditions, nurtured in an environment with proper temperature and humidity, and provided with abundant water and nourishment (25°C, optimal humidity typically at 50%, and a 12-hour dark/light cycle).
Wild animals	This study did not involve wild animals.
Reporting on sex	Both male and female mice from 6 to 10 weeks old were used for this studies.
Field-collected samples	This study did not involve field-collected samples.
Ethics oversight	All animal experiments were first approved by the Laboratory Animal Welfare and Ethics Committee of Zhejiang University (AP CODE: ZJU20230168).

Note that full information on the approval of the study protocol must also be provided in the manuscript.

Plants

Seed stocks	This study does not involve plants.
Novel plant genotypes	This study does not involve plants.
Authentication	This study does not involve plants.

Flow Cytometry

Plots

Confirm that:

☒ The axis labels state the marker and fluorochrome used (e.g. CD4-FITC).

☒ The axis scales are clearly visible. Include numbers along axes only for bottom left plot of group (a 'group' is an analysis of identical markers).

☒ All plots are contour plots with outliers or pseudocolor plots.

☒ A numerical value for number of cells or percentage (with statistics) is provided.

Methodology

Sample preparation	Bone marrow, peripheral blood, and in vitro cell cultures were prepared into a single cell suspension in PBS. Cells were filters (40uM) before FACS.
Instrument	Samples were analyzed using Cytoflex LX (Beckman).
Software	Data were analyzed using CytExpert.
Cell population abundance	Purity of isolated samples was determined by antibody stain and flow cytometry.

Gating strategy

FSC-A/SSC-A for mononuclear cells, FSC-A/FSC-H for singlets, Lineage cocktail (CD3, CD4, CD8,Mac1, Gr-1, B220, CD45R, IgM) for Lin- cells, Lin- Sca1+ c-Kit+ for LSK, Lin- Sca1+ c-Kit+ CD150+ CD48- for LT-HSC, Lin- Sca1+ c-Kit+ CD150- CD48- for ST-HSC, For Lineage analysis, B cells (B220+IgM+), T cells (CD4+ CD8+CD3+) and myeloid cells (Mac1+Gr-1+).

☒ Tick this box to confirm that a figure exemplifying the gating strategy is provided in the Supplementary Information.
